# Neoadjuvant everolimus in renal angiomyolipoma with or without tuberous sclerosis complex: Results from a multicenter, retrospective study

**DOI:** 10.1002/cam4.70181

**Published:** 2024-09-15

**Authors:** Ruopeng Su, Tingxuan Huang, Liangyou Gu, Yige Bao, Zhihong Liu, Pinghong Dao, Lin Yao, Xiaoyi Hu, Guanghou Fu, Jitao Wu, Thibault Tricard, Guangyu Wu, Minfeng Chen, Chancan Li, Zhiyang Huang, Bing Zheng, Yonghui Chen, Wei Xue, Gang Guo, Pei Dong, Jiwei Huang, Jin Zhang

**Affiliations:** ^1^ Department of Urology Ren Ji Hospital, Shanghai Jiao Tong University School of Medicine Shanghai China; ^2^ Department of Urology Oncology Sun Yat‐sen University Cancer Center, State Key Laboratory of Oncology in South China, Collaborative Innovation Center of Cancer Medicine Guangzhou China; ^3^ Department of Urology, The Third Medical Centre Chinese PLA General Hospital Beijing China; ^4^ Department of Urology and Institute of Urology West China Hospital, Sichuan University Chengdu China; ^5^ Department of Urology Xiangya Hospital, Central South University Changsha China; ^6^ Department of Urology First Hospital of Peking University, Institute of Urology, Peking University, National Urological Cancer Center Beijing China; ^7^ Department of Urology Zhongshan Hospital, Fudan University Shanghai China; ^8^ Department of Urology The First Affiliated Hospital, School of Medicine, Zhejiang University Hangzhou China; ^9^ Department of Urology Yantai Yuhuangding Hospital, Qingdao University Yantai Shandong China; ^10^ Department of Urology Nouvel Hôpital Civil, Hôpitaux Universitaires de Strasbourg Strasbourg France; ^11^ Department of Radiology Renji Hospital, Shanghai Jiao Tong University School of Medicine Shanghai China; ^12^ The Department of Urology AnHui NO.2 Provincial People Hospital Hefei China; ^13^ Department of Urology Quanzhou First Hospital affiliated to Fujian Medical University Quanzhou China; ^14^ The Department of Urology The Second Affiliated Hospital of Nantong University Nantong China

**Keywords:** adverse events, everolimus, neoadjuvant, renal angiomyolipomas, treatment response, tuberous sclerosis complex

## Abstract

**Objectives:**

To assess the efficacy and safety of preoperative neoadjuvant everolimus in renal angiomyolipomas (AML) patients with or without Tuberous Sclerosis Complex (TSC).

**Materials and Methods:**

This multi‐institutional retrospective study enrolled renal AML patients who underwent partial nephrectomy (PN) or total nephrectomy after receiving at least 1 month of pre‐operative everolimus. Imaging evaluations were collected before and after treatment, along with demographic, surgical, and follow‐up information. The primary outcome was tumor volume reduction of ≥25%, with additional outcomes including recurrence, perioperative outcomes, renal function, and safety.

**Results:**

From January 2015 to July 2022, 68 renal AML patients were studied—41 with TSC and 27 without. During everolimus treatment, 61.0% (25/41) of TSC patients and 44.4% (12/27) of non‐TSC patients achieved tumor reduction of ≥25%. Additionally, 41.5% (17/41) of TSC patients and 18.5% (5/27) of non‐TSC patients achieved a ≥ 50% reduction. Three TSC patients and 1 non‐TSC patient discontinued treatment due to side‐effects. Most patients (92.7% TSC, 85.2% non‐TSC) underwent PN. After everolimus treatment, the necessary total nephrectomy decreased to 41.2% (7/17) from baseline. Postoperatively, 1 grade 3 and 3 grade 2 complications occurred, with no grade 4 or 5 complications. After a median follow‐up of 24 months, only 1 TSC patient recurred with a diameter >3 cm. Retrospective nature is the major limitation of this study.

**Conclusion:**

Everolimus was effective and well‐tolerated in neoadjuvant treatment for renal AML, especially in TSC patients. This neoadjuvant combination strategy of everolimus and PN could effectively controls recurrence and preserves renal function.

## 1 INTRODUCTION

Angiomyolipomas (AML) is a benign mesenchymal tumor consisting of aberrant thick‐walled blood vessels, smooth muscle cells, and adipose tissue.^1^ AML can arise on its own or, in many cases, in association with the tuberous sclerosis complex (TSC). Indeed, approximately 80% of TSC lesions, which may grow from a common progenitor cell that has both alleles of either TSC1 or TSC2 inactivated, will suffer renal AMLs.^2^ AML symptoms include abdominal pain, a palpable lump, macroscopic hematuria, and hypertension; however, the majority of patients are asymptomatic through to their tumors grow big.^3^ The most concerning complication of AMLs are their proclivity to rupture and bleed spontaneously, with a potential risk of mortality.^4^

The primary objective in patients with renal AMLs is to avoid bleeding and enlargement of AMLs, as well as preserve renal function. The main treatment methods for this disease include active surveillance, surgical resection, embolization, and drug treatment. Mammalian target of rapamycin (mTOR) inhibitors can achieve satisfactory results in treating TSC, which has been recommended as a first‐line treatment option in NCCN guidelines^5^ The efficacy of mTOR inhibitor everolimus in AML was broadly investigated in recent studies with a reduced the AML volume by ≥50% in more than 50% patients with TSC and a ≥25% reduction in tumor volume in 71.4% patients in non‐TSC AML at 6 months of administration.^6^, ^7^ The problem with long‐term everolimus treatment is that it shrinks AMLs but does not cure them, and it raises the chance of developing gonadal dysfunction, interstitial lung disease, and immunosuppression‐related complications.^8^, ^9^

Patients with TSC‐associated AMLs have multifocal and bilateral lesions, as well as a significant recurrence rate when compared to sporadic AMLs. As a result, comprehensive therapy is required for TSC‐associated AML.^10^ Previous case reports indicated that preoperative mTOR inhibitor therapy for TSC‐associated AML may lower the incidence of recurrence and the difficulty of partial nephrectomy (PN).^10^, ^11^ While everolimus exhibits good efficacy in AMLs, the addition of surgery procedure maximizes the possibility of cure and therefore, avoid potential toxicity from long‐term everolimus administration.

For patients with sporadic renal AML who need treatment, embolization and surgery are optional treatment options. Although embolization is more minimally invasive, the drawback was high recurrence rate.^12^ In comparison, PN showed more durable efficacy for long‐term control. The long‐term recurrence rate was only 0%–3% in sporadic AML patients receiving PN.^13^

The relatively high recurrence rate of embolization led to the consideration of surgery treatment. However, it seems challenge to perform PN in large and complex renal AML. To our knowledge, no study reports the outcome of renal AMLs that introduces neoadjuvant therapy to shrink the tumor and create timing for PN leading to a low level of evidence for guidelines in this population. Herein, we conduct a multicenter retrospective study to evaluate the outcomes of neoadjuvant everolimus in renal AMLs with or without TSC.

## 2 MATERIALS AND METHODS

### 2.1 Study population

After the study protocol was approved by the independent institutional review board, we retrieved data from the electronic medical records of renal AML patients from 12 high‐volume centers in China between Jan 2015 and July 2022. Patients ≥18 years who presented with a radiographic diagnosis of an AML ≥3 cm in longest diameter and who were candidates for active surveillance, surgical, or percutaneous intervention were enrolled. The main inclusion criteria include receiving everolimus administration before surgery, identified TSC and non‐TSC, and underwent surgery after neoadjuvant therapy. PN or nephrectomy were accepted. Everolimus was administrated at least for 1 month before surgery. Imaging evaluations were available at baseline or during the neoadjuvant period. The diagnosis of TSC was performed according to clinical guideline, and 19 patients have the results of genetic testing (NGS or exome sequencing).

### 2.2 Study design and treatment

Everolimus was administered 10 mg once daily orally, and based on safety findings, dose modifications were allowed to reduce to 5 mg once daily. Computed tomography (CT) or magnetic resonance imaging (MRI) (same modality used throughout the study for each patient) was performed at baseline and after drug administration. Blood analysis, urine analysis, blood biochemistry, and a chest radiograph were reviewed after treatment to evaluate the effectiveness and safety of the drug. The abdominal image was evaluated every one or 2 months before surgery. At the end of the treatment, a comprehensive preoperative evaluation was conducted to eliminate the definite contraindications.

All patients were examined with CT or MRI before drug treatment, with the greatest tumor diameter >3 cm as the target lesion and other lesions as nontarget lesions. Tumor volume was calculated by radiologists using a standard clinical three‐dimensional image analysis software on CT or MRI images. Primary outcome of this study involved the proportion of patients with a confirmed AML response defined as a ≥25% tumor volume reduction (sum of volumes of all target AMLs identified at baseline) and the absence of AML progression. Other methods, including dynamic contrast‐enhanced MRI and enhanced CT were allowed. To evaluate the feasibility of PN, we collected the conclusions of preoperative assessment before or after everolimus treatment from investigators.

### 2.3 Follow‐up

During follow‐up, patients received CT scan and renal function evaluation every 3–6 month. Adverse events were monitored throughout the study and graded according to the Common Terminology Criteria for Adverse Events v3.0 via patient reported or caregiver‐reported responses as well as investigator assessment.

### 2.4 Surgery approach

Following completion of neoadjuvant therapy, patients underwent PN or total nephrectomy (open or laparoscopic or robot‐assisted at the surgeon's discretion).

### 2.5 Data Collection

Demographic variables and tumor characteristics were collected for baseline characteristics analysis. During drug administration, the imagological examination, Blood biochemistry, and other examination associated with any Adverse events were collected. Perioperative information was recorded including Surgical Approach and Type, blood loss, transfusions, Duration of surgery, length of hospitalization, and complications.

### 2.6 Statistical analysis

Statistical analysis was performed using SPSS version 26.0 and GraphPad Prism 8. Pearson's chi‐square test, Mann–Whithey *U* test or Fisher's exact test was used to comparing categorical variables. The unpaired *t*‐test was used to compare continuous variables. Statistical significance was set at *p* < 0.05.

## 3 RESULTS

### 3.1 Patient characteristics

Between January 2015 and July 2022, 137 patients with AML from 12 academic sites were identified as taking everolimus before surgery. There were 97 TSC‐AML cases and 40 non‐TSC AML cases. Following the screening of inclusion and exclusion criteria, 41 TSC‐AML cases and 27 non‐TSC AML cases were analyzed in this study (Figure S1). The median number of target AMLs for the overall cohort was 2 (range:1–9). As indicated in Table 1, patients in TSC group showed a median age of 34 years (range 18–59) and 78.0% of them were female; similarly, median age in the non‐TSC group was 35 years (23–55), with 77.8% of them being female. There were some discrepancies between the two group. Bilateral kidneys were more frequent in patients (75.6%, 21/41) with TSC‐AML as compared with (37.0%, 10/27) in the non‐TSC group; and 75.6% (31/41) of patients had TSC‐AML with a longest diameter of 8 cm, compared to 55.6% (15/27) in the non‐TSC group. In the TSC group, 43.9% (18/41) of patients reported symptoms, compared to 22.2% in the non‐TSC group.

**TABLE 1:** Patient demographics.

Variables, *n* (%)	TSC group (*N* = 41)	Non‐TSC group (*N* = 27)	*p*‐Value
Age, median (range)	34 (18–59)	35 (23–55)	0.331
Sex			
Male	9 (22.0)	6 (22.2)	0.975
Female	32 (78.0)	21 (77.8)	
Bilateral angiomyolipoma	31 (75.6)	10 (37.0)	0.001
Longest diameter of largest angiomyolipoma lesion			
<4 cm	2 (4.9)	2 (7.4)	0.219
≥4 cm and <8 cm	8 (19.5)	10 (37.0)	
≥8 cm	31 (75.6)	15 (55.6)	
Number of targeted angiomyolipoma lesion (median)			
1	11 (26.8)	17 (63.0)	0.006
2	11 (26.8)	6 (22.2)	
≥3	19 (46.3)	4 (14.8)	
BMI, mean, kg/m^2^	21.4	23.8	
Symptom			
Without	23 (56.1)	21 (77.8)	0.067
With	18 (43.9)	6 (22.2)	

Abbreviation: TSC, tuberous sclerosis complex.

### 3.2 Treatment efficacy

**FIGURE 1:**
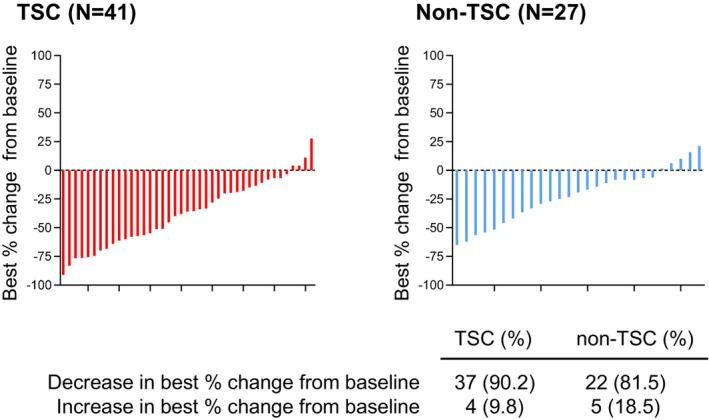
Best percentage change from baseline in the sum of volumes of target AML lesions. Each bar represents one patient.

Table 2 illustrates the volume of change of target lesions. Following a median of 3 months of everolimus administration, overall median rate of tumor shrinkage was 28.6%. Among all patients, 38 of them reached primary outcome with a rate of 55.9%. In cohort analysis, with the median duration of everolimus administration of 4 months for TSC and 3 months for non‐TSC, the primary outcome (≥25% tumor shrinkage) were observed in 61.0% of TSC‐AMLs and 44.4% of non‐TSC AMLs. In addition, 41.5% of TSC‐AMLs and 18.5% of non‐TSC AMLs showed a ≥50% volume reduction. As shown in Figure 1, 90.2% patients in TSC group and 81.5% patients in non‐TSC group showed a decrease of lesion as compared with baseline, respectively. The conclusion of the preoperative evaluations suggested that in 17 patients unfit for PN at baseline, 10 of them (58.8%) transferred to PN after everolimus administration with a median of 41.9% tumor shrinkage. Even if the patients received total nephrectomy, a median of 33.0% tumor shrinkage was observed.

**TABLE 2:** Treatment response to neoadjuvant everolimus.

Variables, *n* (%)	TSC group (*N* = 41)	Non‐TSC group (*N* = 27)	*p*‐Value
Volume decrease of target lesions, median (range), cm^3^	225.4 (−165.4–3554.8)	59.1 (−74.5–735.3)	0.038
Before therapy, median (range), cm^3^	543.8 (16.8–5943.9)	378.3 (12.7–3831.3)	0.056
After therapy	345.7 (4.0–5544.7)	301.0 (9.7–3096.0)	0.262
Reduction rate, median (range)	35.9 (−27.5–91.0)	19.2 (−21.0–65.1)	0.025
Volume change ≥25%, *n* (%)	25 (61.0)	12 (44.4)	0.181
Volume change (cm^3^) ≥50%	17 (41.5)	5 (18.5)	0.048
Drug duration, median (range), month	4 (2–12)	3 (2–8)	0.005

Abbreviation: TSC, tuberous sclerosis complex.

**FIGURE 2:**
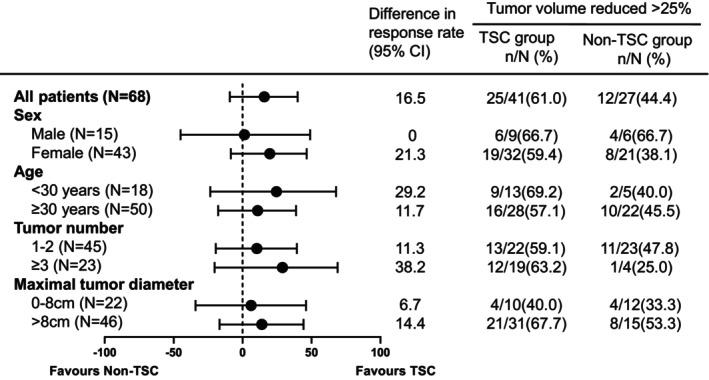
AML response rates by subgroup. 95% CI were obtained from the exact unconditional confidence limits.

Subgroup analysis revealed that independent of gender, age, tumor number, or tumor size, TSC patients receiving everolimus had a deeper response in TSC than that in non‐TSC patients **(**Figure 2 **)**. For the status of recurrence, following a median follow‐up of 24 months, only one patient in TSC‐AML group occurred a >3 cm AML lesion. No other recurrent event was observed.

### 3.3 Perioperative outcomes and adverse events related to neoadjuvant everolimus

The surgical parameters and perioperative complications were shown in Table 3. Most of patients from either TSC group (92.7%) or non‐TSC group (85.2%) underwent PN. Although operating time was significantly longer in TSC group, as compared with non‐TSC subjects, other parameters including selection of surgical approaches, estimated blood loss, length of stay were comparable between two groups. No major intraoperative complication (EAUiaiC≥3) was observed in either group. Inferior vena cava injury (*n* = 1) was observed in the TSC group. The postoperative complications included wound infection, pulmonary embolism, transfusion, and postoperative DSA embolization. No other serious event of complication was observed.

**TABLE 3:** Surgical parameters and perioperative complications in 68 patients receiving everolimus.

Variables, *n* (%)	TSC group (*N* = 41)	Non‐TSC group (*N* = 27)	*p*‐Value
Surgical approach			
Open	24 (58.5)	8 (29.6)	0.035
Laparoscopic	15 (36.6)	14 (51.9)	
Robotic	2 (4.9)	5 (18.5)	
Surgical type			
Partial nephrectomy	38 (92.7)	23 (85.2)	0.320
Total nephrectomy	3 (7.3)	4 (14.8)	
Estimated blood loss, mL, median (range)	150 (10–4000)	150 (50–800)	0.214
Perioperative blood transfusions, *n* (%)	4 (9.8)	2 (7.4)	0.738
Operating time, minutes, median (range)	145 (60–380)	100 (75–290)	0.012
Length of stay, days	5 (2–18)	5 (3–11)	0.962
Intraoperative complications			
Grade 1	1 (2.4)	0 (0.0)	
Grade 2	1 (2.4)	0 (0.0)	
Grade ≥3	0 (0.0)	0 (0.0)	
Postoperative complications highest grade			
1	5 (12.2)	4 (14.8)	
2	2 (4.9)	1 (3.7)	
3	1 (2.4)	0 (0)	

Abbreviation: TSC, tuberous sclerosis complex.

**FIGURE 3:**
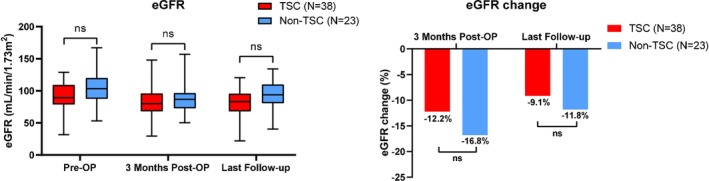
Renal function change before or postoperation, and at the long‐term status.

The change of renal function alteration was also record and shown in Figure 3. As compared with non‐TSC group, there was no significant difference of eGFR in TSC group at pre‐OP, 3 months post‐OP or last follow‐up. When identified the baseline of eGFR as 100%, loss of eGFR was similar between TSC and non‐TSC groups at 3 months post‐OP or last follow up. Furthermore, a slight improvement of renal function was observed in TSC and non‐TSC group post‐OP. The median eGFR change between 3 months post‐OP and last follow‐up was astringed from −12.2% to −9.1% or −16.8% to −11.8% in TSC or non‐TSC group, respectively. Of note, 10 patients in TSC group continued everolimus postoperatively because of contralateral renal AML.

Most common adverse events were similar between TSC and non‐TSC patients, including oral mucositis, maculopapular rash, hypertriglyceridemia, cholesterol high, fatigue, irregular menstruation (female) (Table 4). Gr3‐4 AEs oral mucositis, maculopapular rash, irregular menstruation (female) in TSC and oral mucositis, cholesterol high, irregular menstruation (female) in non‐TSC group were observed. Events leading to discontinuation of treatment occurred in 7.3% of patients in TSC group and 3.7% in non‐TSC group. The reasons for discontinuation were Gr3‐4 AEs and patient intolerance. The surgery was performed 1 week after treatment completement.

**TABLE 4:** Adverse events of any cause.

Variables, *n* (%)	TSC group (*N* = 41)		Non‐TSC group (*N* = 27)	
Adverse event	Any grade	Grade 3–4	Any grade	Grade 3–4
Any event	34 (82.9)	4 (9.8)	41 (74.1)	3 (11.1)
Event leading to discontinuation of treatment	3 (7.3)	3 (7.3)	1 (3.7)	1 (3.7)
Oral Mucositis	29 (70.7)	2 (4.9)	17 (62.9)	1 (3.7)
Maculopapular Rash	5 (12.2)	1 (2.4)	4 (14.8)	0 (0.0)
Hypertriglyceridemia	3 (7.3)	0 (0.0)	2 (7.4)	0 (0.0)
Cholesterol high	3 (7.3)	0 (0.0)	3 (11.1)	1 (3.7)
Fatigue	4 (9.8)	0 (0.0)	2 (7.4)	0 (0.0)
Diarrhea	1 (2.4)	0 (0.0)	0 (0.0)	0 (0.0)
Nausea	1 (2.4)	0 (0.0)	1 (3.7)	0 (0.0)
Vomiting	2 (4.9)	0 (0.0)	1 (3.7)	0 (0.0)
Pneumonitis	1 (2.4)	0 (0.0)	0 (0.0)	0 (0.0)
Irregular menstruation (female)	5 (12.5)	1 (2.4)	4 (19.0)	1 (3.7)
Anemia	2 (4.9)	0 (0.0)	1 (3.7)	0 (0.0)
ALT increase	3 (7.3)	0 (0.0)	1 (3.7)	0 (0.0)
AST increase	2 (4.9)	0 (0.0)	1 (3.7)	0 (0.0)
Neutropenia	1 (2.4)	0 (0.0)	1 (3.7)	0 (0.0)
Fever	1 (2.4)	0 (0.0)	0 (0.0)	0 (0.0)
Elevated Creatinine	1 (2.4)	0 (0.0)	0 (0.0)	0 (0.0)
Upper respiratory infection	2 (4.9)	0 (0.0)	1 (3.7)	0 (0.0)

Abbreviation: TSC, tuberous sclerosis complex.

## 4 DISCUSSION

Our study shows that everolimus was clinically active in the neoadjuvant setting in renal AML patients with 55.9% patients achieving a ≥25% tumor volume reduction. These promising results are particularly important in patients with TSC compared to non‐TSC. 61.0% and 41.5% of TSC‐AMLs patients experienced a ≥25% and a ≥50% volume reduction, respectively, while 44.4% and 18.5% of non‐TSC AMLs showed a ≥25% and a ≥50% volume reduction, respectively. Tolerance was well with a 10.3% rate of grade 3–4 AEs related to systemic treatment and no severe intraoperative and postoperative complication occurring.

Everolimus is now the first‐line option for TSC treatment due to advances in genetic and molecular biology. It can suppress mTOR activity and prevent renal AMLs from enlarging. The EXIST2 trial (Everolimus for AMLs associated with TSC or sporadic lymphangioleiomyomatosis) found that 42% of subjects in the everolimus group had ≥50% reduction of lesion size, in comparison with 0% in placebo group.^6^ A recent study enrolling subjects with sporadic AMLs showed a ≥25% reduction in tumor volume at 4 months in 10/18 subjects and at 6 months in 10/14 subjects.^7^ Although the efficacy of everolimus is remarkable, following analysis of EXIST2 trial indicated that a part of patients underwent regrowth of tumor 48 weeks after everolimus discontinuation.^8^ Two patients in Geynisman, D. M. et al. study also showed notable regrowth at 12 months after demonstrating a ≥50% lesion size reduction at 4 months.^7^ For sporadic AMLs, PN provided long‐term tumor control. But unfortunately, PN may be impossible for patients with large and complex tumors. Although neoadjuvant and pre‐surgical everolimus may shrink primary tumors, no study illustrates the ability of this therapy to facilitate PN in sporadic AML. Therefore, comprehensive treatment with the goal to achieve durable control is attractive for AMLs with or without TSC.

Herein, we performed a multi‐center, retrospective study to explore the feasibility, effectiveness, and practicality of using neoadjuvant everolimus followed by PN for patients with AMLs, regardless of the presence of TSC. The advantages of this strategy include: (1) Neoadjuvant everolimus reduces tumor volume to some extent, lowering the incidence the difficulty of performing PN, while also providing an opportunity for patients who are unable to undergo immediate PN; (2) It provides favorable renal function preservation and achieves durable tumor control with low recurrence rate simultaneously, ensuring the postoperative life quality for these patients; (3) It avoids the need for long‐term administration of everolimus, therefore reduces the risk of late adverse events and potential financial burden.

Everolimus targeting the pathogenesis of AML induced a deep shrinkage of tumor, particularly in TSC‐associated disease. In our study, 90.2% patients in TSC group and 81.5% patients in non‐TSC group showed a decrease of lesion as compared with baseline. More than half of patients in TSC group showed a ≥50% lesion size reduction. The deep response of TSC tumor indicated a strong sensitivity for everolimus, which may suggest a dominant role of mTOR pathway in TSC tumor development. The primary outcomes of this study were comparable with EXIST2 trial and Geynisman, D. M. et al. study.^6^, ^7^ Indeed, many patients with renal AMLs were identified as unable or hard to perform a PN with the causes such as technically impossible in large confluent lesions, or uncertain malignancy. Initial surgery and embolization usually fail in up to 20% of TSC‐related AML tumors, which leads to the requirement of repeat surgery or embolization and restricts their value in the treatment of TSC‐AML.^14^, ^15^ In this scenario, neoadjuvant everolimus with high efficacy in inducing AML shrinkage will set the stage for surgery. Our observation further confirmed this concept that 10 of 17 patients unfit for PN at baseline were transferred to PN after 3 to 4 months of everolimus administration. Overall, preoperative everolimus facilitated the feasibility of PN and simplified operation difficulty.

A decline in renal function has been reported as the natural history of renal AML patients with TSC.^12^, ^16^ Either PN or embolization alone led to the inevitable loss of renal function. The embolization restricts the acquirement of histologic diagnosis. Unilateral nephrectomy results in substantial loss of renal function. In comparison, PN provides unequivocal management of AML for histologic confirmation. Moreover, dynamic analysis for eGFR suggested neoadjuvant everolimus induced limited loss of ΔeGFR, and renal function maintained well for a long‐term. Another issue for embolization is the potential high recurrence rate, particularly in AML with TSC.^12^ In our retrospective cohort, only one of 97 patients experienced recurrence in median follow‐up of 24 months.

Although the efficacy and safety of everolimus has been confirmed, the challenges remained in some situations. mTOR inhibitors have potent antiproliferative and immunosuppressive effects, causing their usage to be with dose interruptions or adjustments. And after discontinuation of everolimus, 31.3% of patients experienced regrowth of AML.^8^ Indeed, late AE management and the risk of regrowth contribute to expense increases and AE suffering. Many patients take personal values and preferences/individual situations into the consideration of treatment selections. For examples, the medical institution is far away from home, the expense of treatment is high, and the patient expects to be durable control in a defined course. Neoadjuvant everolimus followed by PN will be considered in these situations. In our respective study, most patients received PN after three or 4 months of everolimus. Consistently, EXIST2 trials show a median time to AML response of 2.9 months.^6^, ^17^ All the data above indicated that neoadjuvant everolimus could create a chance for surgery within a stable time pried.

The major limitation of our study was the small sample size, which was attributable to the rarity of AML with or without TSC. Otherwise, with the nature of retrospective study, we only just collected the information from investigator's perspective to identify whether patients were fit for PN. Nonetheless, our analysis provides the largest series and unique perspective to identify the clinical value of neoadjuvant everolimus in AML.

## 5 CONCLUSION

In conclusion, neoadjuvant everolimus followed by PN is an attractive option for AML patients with high efficacy, maximized renal function preservation and a low burden of long‐term management.

## AUTHOR CONTRIBUTIONS

**Jiwei Huang:** Conceptualization (equal); funding acquisition (equal); investigation (equal); supervision (equal); validation (equal); writing – original draft (equal). **Ruopeng Su:** Data curation (equal); formal analysis (equal); investigation (equal); methodology (equal); writing – original draft (equal). **Tingxuan Huang:** Data curation (equal); formal analysis (equal); investigation (equal); writing – original draft (equal). **Liangyou Gu:** Data curation (equal); formal analysis (equal); investigation (equal); writing – original draft (equal). **Zhihong Liu:** Data curation (equal); formal analysis (equal); investigation (equal); writing – original draft (equal). **Pinghong Dao:** Data curation (equal); formal analysis (equal); investigation (equal); writing – original draft (equal). **Lin Yao:** Data curation (equal); formal analysis (equal); investigation (equal); writing – original draft (equal). **Xiaoyi Hu:** Data curation (equal); formal analysis (equal); investigation (equal); writing – original draft (equal). **Guanghou Fu:** Data curation (equal); formal analysis (equal); investigation (equal); writing – original draft (equal). **Jitao Wu:** Data curation (equal); formal analysis (equal); investigation (equal); writing – original draft (equal). **Thibault Tricard:** Investigation (equal); methodology (equal); validation (equal). **Guangyu Wu:** Formal analysis (equal); investigation (equal); methodology (equal). **Minfeng Chen:** Conceptualization (equal); supervision (equal). **Chancan Li:** Data curation (equal); formal analysis (equal); investigation (equal); writing – original draft (equal). **Zhiyang Huang:** Data curation (equal); formal analysis (equal); investigation (equal); writing – original draft (equal). **Bing Zheng:** Data curation (equal); formal analysis (equal); writing – original draft (equal). **Yonghui Chen:** Conceptualization (equal); investigation (equal); methodology (equal); supervision (equal). **Wei Xue:** Conceptualization (equal); project administration (equal); supervision (equal). **Yige Bao:** Project administration (equal); supervision (equal); validation (equal). **Gang Guo:** Project administration (equal); supervision (equal). **Pei Dong:** Methodology (equal); project administration (equal); supervision (equal); validation (equal). **jin zhang:** Formal analysis (equal); investigation (equal); project administration (equal); supervision (equal); validation (equal).

## FUNDING INFORMATION

This study was supported by Shanghai Science and Technology Commission Research Project (21ZR1438900), Basic Oncology Research Program from Bethune Charitable Foundation (BCF‐NH‐ZL‐20201119‐024), Wu Jieping Medical Foundation (320.6750.2022‐19‐92), and the Incubating Program for Clinical Research and Innovation of Renji Hospital (PYXJS16‐008, PYIII20‐07).

## CONFLICT OF INTEREST STATEMENT

The authors report no conflict of interest.

## ETHICS STATEMENT

This study was conducted in accordance with the ethical standards of the Declaration of Helsinki and approved by ethics committee of Ren Ji Hospital and all other participating institutions (KY2021‐102). The data was retrieved from electronic medical records of 11 participating institutions and informed consent was obtained from the participants involved.

## Supporting information


Figure S1.

## Data Availability

Jiwei Huang had full access to all the data in the study and takes responsibility for the integrity of the data and the accuracy of the data analysis. The data that support the findings of this study are available from the corresponding authors upon request.
