# Supplementary figures and images for: Neoadjuvant everolimus in renal angiomyolipoma with or without tuberous sclerosis complex: Results from a multicenter, retrospective study
Source: Cancer Med. 2024 Sep 15;13(17):e70181. doi: 10.1002/cam4.70181 (PMC11403123; doi:10.1002/cam4.70181)

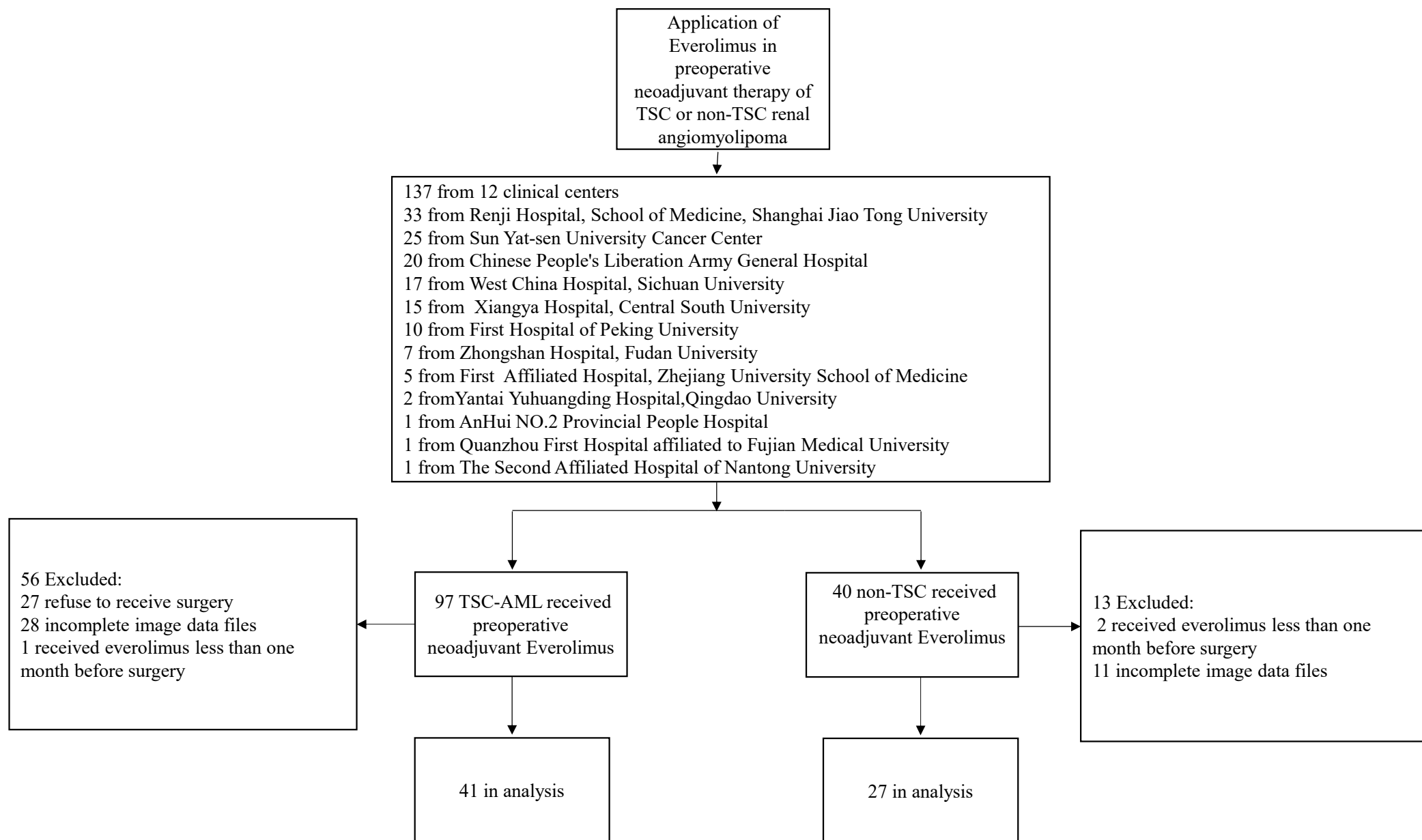

Supplement: Supplementary file 1 — Figure S1. [file CAM4-13-e70181-s001.pdf]
